# Effect of Pre-emptive Injection-based Corticosteroid Administration and Its Routes on Post-Endodontic Pain: A Systematic Review and Meta-analysis of Randomized Clinical Trials

**DOI:** 10.4317/jced.64027

**Published:** 2026-04-25

**Authors:** Reem Al-Yamoor, Stefan Vasile Stefanescu, Maria Miheala Iuga, Fernando Mauricio Espada-Salgado, Steve Bilsborrow, Kavalipurapu Venkata Teja, Kaligotla Apoorva Vasundhara

**Affiliations:** 1 Chester Cosmetic and Dental Care, Chester CH21JF, UK; 2 Top Smiles, Liverpool L6 4DU, Merseyside, UK; 3 Faculty of Health Sciences, Department of Dentistry, National University Jorge Basadre Grohmann, Tacna, Peru; 4 Faculty of Health Sciences, Private University of Tacna, Tacna, Peru; 5 Newtown Dental Center, 161 Cambridge Rd, St Helens WA10 4HA, UK; 6 Department of Conservative Dentistry and Endodontics, Malla Reddy Institute of Dental Sciences, Malla Reddy Vishwavidyapeeth, Hyderabad, Telangana, India; 7 Department of Prosthodontics, Saveetha Dental College and hospitals, Saveetha Institute of Medical and Technical Sciences, Saveetha University, Chennai, TamilNadu, India

## Abstract

**Background:**

Post-endodontic pain PP is a frequent complication following root canal treatment RCT, primarily resulting from acute inflammation. Pre-emptive corticosteroid administration has been proposed to attenuate this inflammatory response and reduce PP. This systematic review and meta-analysis assessed the effectiveness of injection-based corticosteroids delivered through different routes in minimizing PP at various postoperative intervals.

**Material and Methods:**

A comprehensive search of PubMed, Scopus, Web of Science, LILACS, Cochrane Library, and Google Scholar was conducted up to March 2025, following PRISMA 2020 guidelines. Randomized controlled trials evaluating pre-emptive corticosteroid injections via infiltrative, intraligamentary, or intramuscular routes were included. Risk of bias was assessed using the Cochrane RoB 2.0 tool, and meta-analyses were performed using Review Manager 5.4. The review was prospectively registered in PROSPERO CRD420251005886.

**Results:**

Nine RCTs comprising 917 participants were analyzed. Corticosteroid injections, including dexamethasone, methylprednisolone, and betamethasone, significantly reduced PP compared with placebo at all time points, with the most pronounced effect within the first 12 hours and sustained, though reduced, benefits up to 72 hours. Infiltrative injections demonstrated greater efficacy than intraligamentary routes.

**Conclusions:**

Pre-emptive corticosteroid injections, particularly dexamethasone via infiltrative or intraligamentary routes, effectively reduce post-endodontic pain, especially within the first 24 hours.

## Introduction

Pain is a common and often distressing complication following root canal treatment, affecting patient comfort and clinical satisfaction. Although typically self-limiting, moderate to severe pain within the first 48-72 hours can significantly impact patient perception of treatment success (1). The etiology of PP is multifactorial, primarily driven by an acute inflammatory response to mechanical, chemical, or microbial insults during instrumentation (2). Key inflammatory mediators such as prostaglandins, bradykinin, histamine, and substance P are released from injured periapical tissues, contributing to hyperalgesia and nociceptive signalling. Additionally, patient-specific factors, such as preoperative pain levels and emotional stress, further modulate pain perception (2). Given the multifaceted nature of endodontic pain, pharmacological interventions and intracanal medication play a critical role in its management (3). Systematic reviews (4),(5) have explored the efficacy of analgesics, steroids in controlling PP pain. Corticosteroids, in particular, have been shown to inhibit the secretion of such as prostaglandins, leukotrienes, and bradykinin, which are regarded as the key inflammatory mediators (5). Additionally, preemptive drug administration significantly reduces pain perception during treatment and helps minimize intraoperative and PP pain (6). Corticosteroids, recognized for their potent anti-inflammatory properties, have been extensively investigated in this context. A recent review data (7), found that corticosteroids significantly reduced postoperative endodontic pain at 6, 12, and 24-hours post-treatment. Similarly, Jalalzadeh et al. (8) reported decreased post-endodontic pain levels with single oral dose of prednisolone prescribed priorly. The route of corticosteroid administration may play a crucial role in its effectiveness for pain management. Injection-based administration, including infiltrative, intraligamentary, and intramuscular injections, allows for targeted drug delivery and may enhance pain control (9). However, there is still no clear consensus on the most effective route. A recent systematic review by Alajlan et al. (10) highlighted that preemptive systemic corticosteroids improve the enhanced anaesthetic success in pulpal inflammation, emphasizing their potential benefits in pain management. Systematic reviews have also assessed the efficacy of preemptive corticosteroid administration in reducing postoperative pain (3 , 5 , 11). However, there is currently no conclusive evidence to determine whether pre-emptive corticosteroids administered via injection-based routes alone are effective in minimizing post-endodontic pain. Although, previous systematic reviews (3 , 5 , 11), have explored the efficacy of corticosteroids in PP control, there is a significant heterogeneity that exists regarding drug choice, dosage, timing, and route of administration. The comparative effectiveness of these routes remains unclear, and no standard clinical protocol has been established. Given the variability in study designs, corticosteroid types, dosages, and administration routes, a comprehensive synthesis of the available evidence is essential. Therefore, the aim of this systematic review and meta-analysis is to evaluate the effects of pre-emptive injection-based corticosteroid administration routes in PP reduction.

## Material and Methods

**Figure 1:**
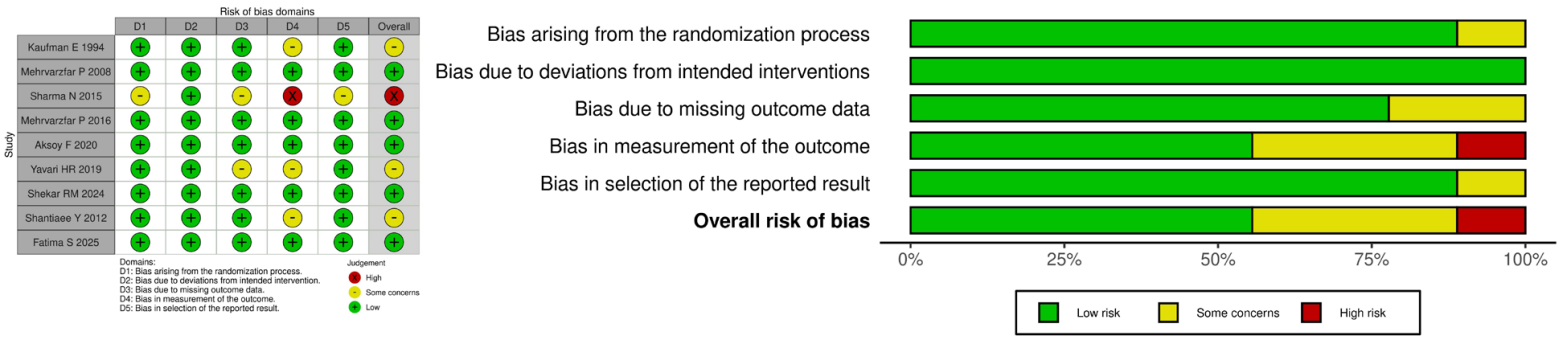
a) Risk of bias graph, b) Risk of bias summary.

The review was registered in PROSPERO CRD420251005886, before the commencement of the detailed analysis. PRISMA 2020 guidelines were followed for drafting the current review. Focused Question This review aimed to determine the effectiveness of pre-emptive corticosteroid administration delivered through various injection-based routes in reducing post-endodontic pain. The structured research question was: "In adult patients undergoing root canal treatment P, how does pre-emptively administered corticosteroid via injection techniques, including infiltrative, intraligamentary, or intramuscular routes I, compared with placebo C, influence the reduction of postoperative endodontic pain O as evaluated in randomized clinical trials S?" Eligibility Criteria: Inclusion Criteria Randomized clinical trials RCTs Studies evaluating pre-emptive corticosteroid administration via injection-based routes intraligamentary, intramuscular, infiltrative Root canal treatment performed in adult patients Postoperative pain reported as the primary outcome Pain assessment conducted using a validated tool Exclusion Criteria Animal studies or in vitro studies Review articles, case reports, or case series Observational or non-randomized studies Studies that: o Did not involve pre-emptive corticosteroid administration o Did not assess postoperative endodontic pain o Did not report clear outcome data o Lacked sufficient methodological details Search Strategy A comprehensive and systematic search was conducted across six major electronic databases, including PubMed, Scopus, EMBASE, LILACS, Web of Science, and Google Scholar, from their inception until March 2025. Additionally, the Cochrane Library was searched for any relevant systematic reviews or clinical trials Figure 1.

1

The search strategy was constructed using a combination of MeSH terms, free text words, and Boolean operators related to root canal treatment, pre-emptive corticosteroid administration, and postoperative pain. The search was not restricted by the date of publication but was limited to the English language. A hand search of the reference lists of included studies was performed to identify additional relevant articles. Furthermore, grey literature was explored in repositories such as the Open Access Theses and Dissertations OATD, Networked Digital Library of Theses and Dissertations NDLTD, and DART-Europe E-theses Portal DEEP to minimize publication bias. The detailed search strategy for each database is presented in Table 1.

Table 1

Duplicates were removed using Zotero reference management software. Selection of Studies Two independent reviewers MML and KAV conducted the study selection in two stages. In the first stage, the titles and abstracts of all retrieved articles were screened independently to identify potentially relevant studies. In the second stage, the full texts of these selected articles were obtained and assessed in detail against the predefined inclusion and exclusion criteria. Any disagreements between the two reviewers were resolved through discussion, and a third reviewer KVT was consulted when consensus could not be reached. To evaluate the reliability of the selection process, inter-reviewer agreement was calculated using Cohen's Kappa , which yielded a substantial agreement of 0.81. Data Collection and Extraction Data extraction was independently performed by two reviewers MML and KAV using a pre-designed and standardized data extraction form. Participant-related variables collected included sample size, age range, gender distribution, and preoperative diagnosis. Detailed intervention characteristics were also recorded, such as the route of corticosteroid administration, type and dosage of corticosteroid, volume of the drug administered, and specifications of the syringe and needle used. Primary outcome data included postoperative pain intensity at multiple time intervals, pain assessment tools utilized, and the duration of follow-up. Additional outcomes such as postoperative analgesic consumption were also extracted where available. When data were missing or unclear, attempts were made to contact the corresponding authors for clarification. Any discrepancies between reviewers were resolved through discussion, and a third reviewer KVT was involved to achieve consensus where necessary. Risk of Bias Assessment The risk of bias for the included studies was independently assessed by two reviewers MML and KAV using the Cochrane Risk of Bias 2.0 RoB 2.0 tool, following the recommended criteria for randomized controlled trials. Each domain, including the randomization process, deviations from intended interventions, missing outcome data, measurement of the outcome, and selection of the reported result, was evaluated and rated accordingly. The overall risk of bias for each study was then determined. A third reviewer KVT was consulted to resolve any disagreements and to ensure accuracy and consistency in the assessment process. Meta-analysis: Meta-analysis using Review Manager 5.4 revealed a significant reduction in post-endodontic pain intensity in patients receiving corticosteroids compared to placebo across all time intervals. The pooled analysis showed a consistent effect favouring corticosteroids, with the greatest pain reduction observed at 72 hours mean difference: -2.07; 95% CI: -2.33 to -1.81 and 6 hours -0.86; 95% CI: -0.97 to -0.75. At 24 hours, subgroup analysis by route of administration showed that infiltrative injections had a larger effect size -2.57; 95% CI: -2.90 to -2.23 than intraligamentary injections -1.10; 95% CI: -1.42 to -0.78, although both routes significantly reduced pain. Overall, corticosteroid use was consistently associated with significantly lower pain scores than placebo at all evaluated time points, despite high heterogeneity across studies.

## Results

**Figure 2:**
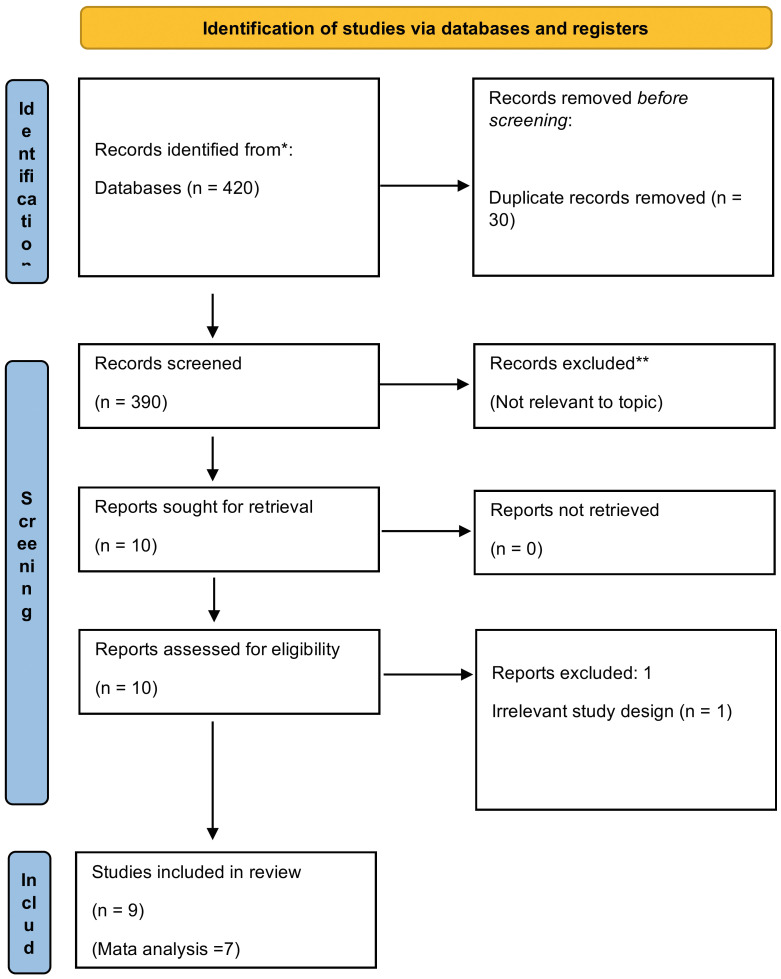
PRISMA Flow Chart.

A total of 420 records were identified through systematic electronic database searches. The number of records retrieved from each source was as follows: PubMed n = 12, Scopus n = 11, Cochrane Library n = 18, EMBASE n = 19, Web of Science n = 23, and Google Scholar n = 337. No additional studies were identified through hand-searching or clinical trial registries. After removing 30 duplicates, 390 records were screened. Following the title screening, 373 articles were excluded, and after abstract evaluation, 7 additional articles were excluded. Ten full-text articles were assessed for eligibility, of which one study was excluded because it did not include an appropriate control group for comparison (12). Consequently, nine studies (13 - 21) were included in the present review, as depicted in the PRISMA 2020 flow diagram Flow Chart 1, (Fig. 2).

2

Study Characteristics Nine studies (13 - 21) with a combined total of 917 participants were included, with sample sizes ranging from 45 (13) to 242 (18). Participant ages varied between 18 years (15) and 71 years (13). Most studies included patients with symptomatic irreversible pulpitis (13 - 21), while a few also involved symptomatic apical periodontitis (13 , 20 , 21). Dexamethasone was the most frequently used corticosteroid (13 - 21) and was administered through intraligamentary, intramuscular, or infiltration routes. Betamethasone (18) and methylprednisolone (13) were used in some studies. The drug volume, delivery technique, and needle type varied among trials Table 2.

Table 2

Most studies (15 , 16 , 18 , 19 , 21) administered local anesthesia lidocaine 2% with epinephrine 1:100,000 before corticosteroid injection, while one used articaine (17). In a few studies (13 , 14 , 20), the anesthetic used was not specified. Pain Reduction Outcomes All included studies (13 - 21) assessed postoperative pain using different scales. The Visual Analogue Scale VAS was the most common, (13 - 16 , 18 , 20 , 21) while one used the verbal rating scale (19) and another the Heft-Parker scale (17). Assessment times ranged from 4 hours (20) to 7 days (18). Overall, all studies reported lower post-endodontic pain levels in the corticosteroid groups compared with control or placebo groups Table 3.

Table 3

**Figure 3:**
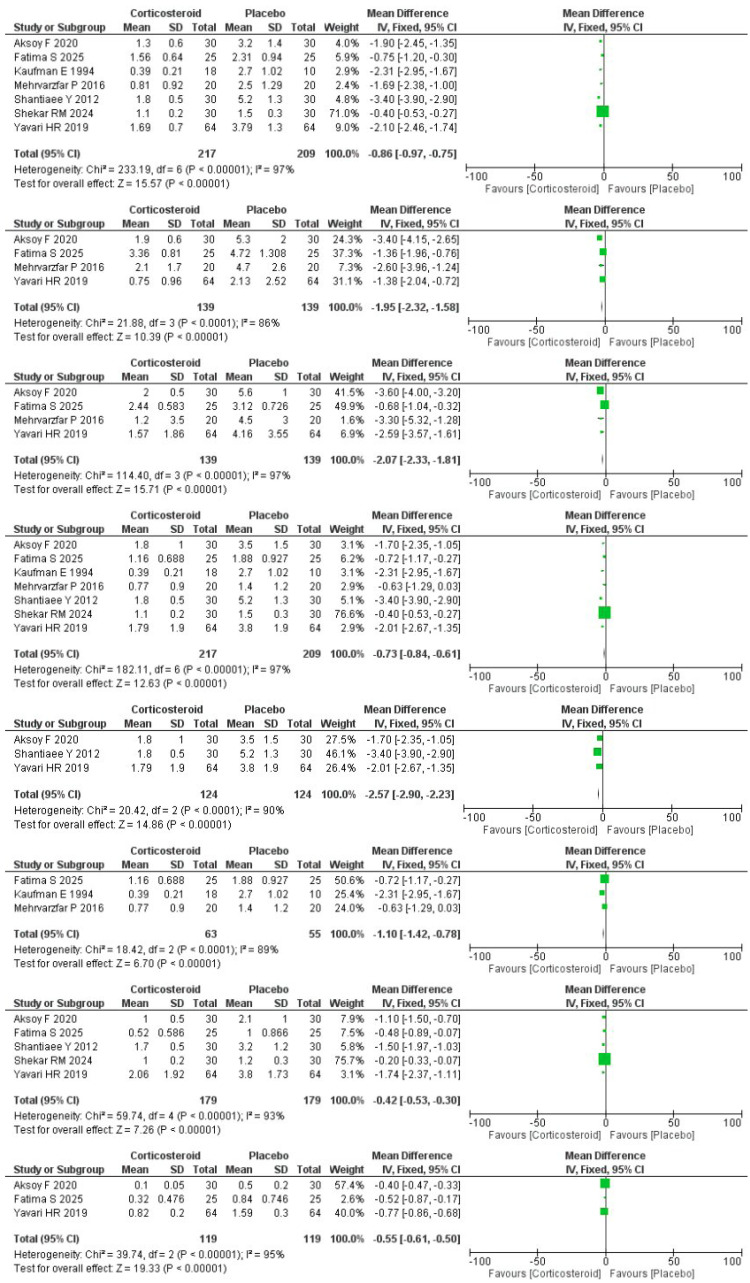
a: Forest plot depicting the overall pain relief between the interventions, b: Forest plot depicting the pain relief at 6 hours, c: Forest plot depicting the pain relief at 12 hours, d: Forest plot depicting the pain relief at 24 hours , e: Forest plot depicting the subgroup infiltrative route at 24 hours, f: Forest plot depicting the subgroup intraligamnetary route at 24 hours, g: Forest plot depicting the pain relief at 48 hours, h: Forest plot depicting the pain relief at 72 hours.

Pain Reduction at Different Time Intervals Early postoperative pain 0-6 hours: Five studies (14 , 15 , 17 , 21 , 23) reported significant reductions in pain scores in patients receiving corticosteroids compared with placebo. Mehrvarzfar et al. (14) found that only 8% of patients in the dexamethasone group reported pain at 6 hours compared with 40% in the placebo group. Aksoy et al. (17) observed a median pain score of 32 mm in the dexamethasone group versus 90 mm in the placebo group using the Heft-Parker scale. Fatima et al. (21) reported mean VAS scores of 3.36 ± 0.81 in the dexamethasone group and 4.72 ± 1.31 in the placebo group. Similar reductions were also observed by Sharma et al. (15) and Mehrvarzfar et al. (16) confirming that corticosteroids provided superior early analgesia. Pain reduction at 12-24 hours: All five studies above continued to show significantly lower pain in corticosteroid groups. Mehrvarzfar et al. (14) reported pain in only 2% of dexamethasone patients at 12 hours compared with 34% in placebo. At 24 hours, the incidence of pain was 6% in the corticosteroid group and 24% in the placebo group. Aksoy et al. (17) reported median pain scores of 18 mm for dexamethasone and 35 mm for placebo. Sharma et al. (15) observed an 89.5% reduction in pain at 24 hours in the infiltrative corticosteroid subgroup, compared with a 61.7% improvement in the placebo group. Fatima et al. (21) found significantly lower scores at 24 hours for dexamethasone 1.16 ± 0.69 compared with placebo 1.88 ± 0.93. Pain reduction at 48-72 hours: Four studies (16 , 17 , 18 , 21), evaluated pain beyond 24 hours and reported continued benefit with corticosteroids. Aksoy et al. (17) recorded mean pain scores of 10 mm and 1 mm at 48 and 72 hours in the dexamethasone group, compared with 21 mm and 5 mm in the placebo group. Yavari et al. (18) noted a reduction from 5.21 at baseline to 0.82 at 72 hours in the dexamethasone group, compared with 1.59 in placebo. Fatima et al. (21) reported mean scores of 0.32 ± 0.48 and 0.84 ± 0.75 at 72 hours for dexamethasone and placebo, respectively. Pain reduction at 7 days: Two studies (18 , 19) followed participants up to 7 days. Yavari et al. (18) reported a final mean VAS of 0.64 in the dexamethasone group versus 0.93 in placebo. S0ekhar et al. (19) also noted consistently lower pain scores with dexamethasone from 4 hours to 48 hours, with minimal pain by day seven. These results suggest both short-term and sustained analgesic effects of corticosteroids following endodontic treatment. Comparison Between Routes of Administration Infiltration: Aksoy et al. (17) reported that infiltrative dexamethasone produced lower pain scores at all time intervals compared with placebo 32 mm vs 90 mm at 6 hours. Mehrvarzfar et al. (14) found similar findings, with 8% of dexamethasone patients experiencing pain at 6 hours compared with 40% in the placebo group, and only 6% versus 24% at 24 hours. Yavari et al. (18) compared dexamethasone, betamethasone, and placebo and found dexamethasone consistently produced the lowest scores at all intervals, including 1.57 versus 4.16 at 12 hours and 0.64 versus 0.93 at 7 days. Shantiaee et al. (20) reported mean scores of 1.8 for dexamethasone versus 5.2 for placebo at 24 hours. Intraligamentary: Mehrvarzfar et al. (16) demonstrated significant pain reduction following intraligamentary dexamethasone compared with placebo, with mean pain scores of 35.25 mm versus 80 mm at 6 hours. Fatima et al. (21) found similar trends with scores of 3.36 versus 4.72 at 6 hours, which remained significantly lower up to 72 hours. Kaufman et al. (13) reported that 22% of patients receiving methylprednisolone via the intraligamentary route experienced pain at 24 hours, compared with 50% and 76% in active and passive placebo groups, respectively. Intramuscular: Sekhar et al. (19) observed greater pain reduction with intramuscular dexamethasone compared with placebo 1.2 vs 2.0 at 4 hours, with the effect lasting up to 48 hours. Sharma et al. (15) also found that intramuscular dexamethasone reduced pain more effectively than placebo, though infiltrative administration produced faster relief at 12 and 24 hours. Analgesic Consumption Five studies (13 , 14 , 17 , 19 , 21) reported a reduction in additional analgesic use among corticosteroid-treated patients. Kaufman et al. (13) found that only 22% of participants in the methylprednisolone group required rescue analgesics compared with 76% in the placebo group. Aksoy et al. (17) also reported that patients receiving dexamethasone needed fewer postoperative pain medications. Risk of Bias The risk of bias was evaluated using the Cochrane Risk of Bias 2.0 tool. Most studies (14 , 16 , 17 , 19 , 21) showed a low risk of bias across key domains, including randomization, deviations from intended interventions, and outcome measurement. One study (15) had a high risk due to measurement bias. A few (13 , 18 , 20) were rated as having "some concerns," mainly due to missing or unclearly reported data. Overall, the methodological quality of included studies was considered acceptable Figure 1. Meta-analysis of Post-Endodontic Pain Overall pain reduction: Pooled analysis from seven (13 , 16 - 20) randomized clinical trials showed significant reductions in postoperative pain favouring corticosteroid use. The standardized mean difference was -2.17 95% CI: -2.75 to -1.59, p &lt; 0.00001, I² = 78% Figure 3.

3

At 6 hours: Four studies demonstrated significant benefit with a pooled mean difference of -2.07 95% CI: -2.33 to -1.81, p &lt; 0.00001, I² = 97% Figure 3. At 12 hours: Four studies (16 , 17 , 18 , 21) showed continued pain reduction, with a pooled mean difference of -1.95 95% CI: -2.32 to -1.58, p &lt; 0.00001, I² = 97% Figure 3. At 24 hours: Seven studies (13 , 16 - 21) reported a pooled mean difference of -0.73 95% CI: -0.84 to -0.61, p &lt; 0.00001, I² = 97%, confirming sustained analgesic benefit Figure 3. Subgroup analysis 24 hours: For infiltrative corticosteroid injections, the pooled mean difference was -2.57 95% CI: -2.90 to -2.23, I² = 90% Figure 3. Intraligamentary injections showed a mean difference of -1.10 95% CI: -1.42 to -0.78, I² = 89% Figure 3. At 48 hours: Five studies (17 - 21) demonstrated continued pain reduction with a mean difference of -0.55 95% CI: -0.61 to -0.50, p &lt; 0.00001, I² = 95% Figure 3. At 72 hours: Three studies (17 , 18 , 21) showed a pooled mean difference of -0.42 95% CI: -0.53 to -0.30, p &lt; 0.00001, I² = 93%, confirming lasting analgesic effects up to 3 days post-treatment Figure 3. Summary Across all evaluated intervals from 6 to 72 hours, corticosteroid administration resulted in statistically significant reductions in post-endodontic pain compared with placebo. The most pronounced effects were observed within the first 12 hours and remained clinically meaningful up to 72 hours. Subgroup analysis showed that infiltrative injections produced greater pain relief than intraligamentary administration. Despite high heterogeneity due to differences in drug types, doses, and protocols, all studies indicated a consistent direction of effect. These findings support the use of pre-emptive corticosteroid injections, particularly dexamethasone, as an effective strategy for managing post-endodontic pain.

## Discussion

This systematic review and meta-analysis synthesized evidence from nine randomized controlled trials (13 - 21) evaluating the effect of pre-emptive corticosteroid injections on post-endodontic pain. The results demonstrate that corticosteroid administration, primarily dexamethasone, via infiltrative, intraligamentary, or intramuscular routes significantly reduces postoperative pain at multiple time intervals, particularly within the first 24 hours. These findings align with and extend those of previous systematic reviews (3 , 5 , 9 , 22) which also reported favorable analgesic outcomes following corticosteroid use in endodontic therapy at first 24 hours. The biological plausibility of these outcomes is grounded in the anti-inflammatory mechanism of corticosteroids. By inhibiting phospholipase A2 and the subsequent arachidonic acid cascade, corticosteroids suppress the production of pro-inflammatory mediators such as prostaglandins, leukotrienes, and cytokines. These mediators are known to play a pivotal role in the pathogenesis of post-instrumentation flare-ups and hyperalgesia. The consistent reduction in pain scores across all time points in this review reinforces the potential of corticosteroids to attenuate the early inflammatory response induced by endodontic procedures. Among the various administration routes, infiltrative injection was associated with the greatest magnitude of pain relief at 24 hours MD -2.57; 95% CI: -2.90 to -2.23, outperforming intraligamentary injection MD -1.10; 95% CI: -1.42 to -0.78. This is consistent with previous systematic review (23), which suggested that localized delivery of corticosteroids may enhance their efficacy by concentrating the drug at the site of tissue injury. Intramuscular administration, although less effective in immediate pain relief, provided sustained analgesia up to 48 to 72 hours, possibly due to delayed systemic absorption (15 , 19). Importantly, corticosteroid use was also associated with reduced consumption of rescue analgesics. This finding has been corroborated by Suneelkumar et al (5). and Alajlan et al (22). and thus have a clinical significance, particularly for patients with contraindications to NSAIDs or those at risk of gastrointestinal or renal complications (8 , 13 , 17). Furthermore, the improved pain control observed in corticosteroid groups may enhance patient satisfaction and reduce emergency visits after treatment. Recent systematic review also reported the safety with least adverse effect on intraoral administration of injection based corticosteroids (24). Despite these encouraging findings, heterogeneity among studies was considerable I² &gt; 90%. This was attributed to variability in corticosteroid type, dosage, route, and pain assessment scales. These methodological differences limit direct comparison and underscore the need for standardized clinical protocols. In addition, a recent systematic review (25) reported a limited data on the potential adverse effects, thereby limiting conclusions regarding the safety profile of corticosteroid use in endodontics. In comparison with earlier reviews (22 , 23), the present analysis uniquely focuses only on the injection-based corticosteroid delivery. This offers more targeted insights for clinical application. While recently published review (22) included oral or systemic administration, this review narrows the focus to parenteral techniques that allow localized, pre-emptive intervention. This approach is increasingly relevant in minimally invasive and patient-centred endodontics. The important limitation of the present review is the lack of sufficient studies directly comparing different corticosteroid delivery routes. Due to the limited number of such comparative trials, this meta-analysis synthesized results from separate studies based on individual routes of administration. Consequently, the comparisons between delivery methods infiltrative, intraligamentary should be interpreted with caution, as they were not derived from head-to-head trials but from indirect analyses. Future well-designed randomized controlled trials directly comparing these administration routes are essential to validate and strengthen the current findings. Future studies should focus on standardizing corticosteroid regimens, including drug type, dosage, and timing of administration. Long-term safety data, particularly in medically compromised patients, are essential to establish risk profiles. Comparative trials assessing different corticosteroid agents, such as betamethasone versus dexamethasone, and their efficacy when combined with non-opioid analgesics may further enhance clinical strategies. Moreover, studies exploring the impact of corticosteroid use on the success of local anaesthesia and patient-reported outcomes are warranted.

## Conclusions

The findings of this systematic review and meta-analysis demonstrate that pre-emptive corticosteroid injections, particularly when administered through the infiltrative and intraligamentary routes, significantly reduce post-endodontic pain, with the greatest analgesic effect observed within the first 24 hours. Infiltrative administration of dexamethasone showed the highest magnitude of pain reduction among the analysed routes. However, due to the limited number of studies and lack of direct comparisons between different administration routes, these findings should be interpreted cautiously. Further randomized clinical trials are warranted to directly compare various delivery methods and to establish standardized corticosteroid regimens for optimal pain management in endodontics.

## Figures and Tables

**Table 1:** Search Strategy.

Database	Search Terms
PubMed	Root canal treatment OR Endodontic treatment OR Symptomatic irreversible pulpitis AND Preemptive OR Corticosteroids AND Intraligamentary injection OR Intramuscular injection OR Supraperiosteal injection OR Submucosal injection OR Periodontal ligament injection AND Postendodontic pain OR Postobturation pain
Scopus	TITLE-ABS-KEY "Root canal treatment" OR "Endodontic treatment" OR "Symptomatic irreversible pulpitis" AND TITLE-ABS-KEY "Preemptive corticosteroids" OR "Intraligamentary injection" OR "Intramuscular injection" OR "Supraperiosteal injection" OR "Submucosal injection" OR "Periodontal ligament injection" AND TITLE-ABS-KEY "Postendodontic pain" OR "Postobturation pain"
Cochrane Library	Root canal treatment AND Preemptive corticosteroids AND Postoperative pain
EMBASE	'root canal treatment'/exp OR 'endodontic treatment'/exp OR 'symptomatic irreversible pulpitis'/exp AND 'preemptive therapy'/exp OR 'corticosteroids'/exp OR 'intraligamentary injection'/exp OR 'intramuscular injection'/exp OR 'supraperiosteal injection'/exp OR 'submucosal injection'/exp OR 'periodontal ligament injection'/exp AND 'postendodontic pain'/exp OR 'postobturation pain'/exp
Google Scholar	Allintitle: "Root canal treatment" OR "Endodontic treatment" OR "Symptomatic irreversible pulpitis" AND "Preemptive corticosteroids" OR "Intraligamentary injection" OR "Intramuscular injection" OR "Supraperiosteal injection" OR "Submucosal injection" OR "Periodontal ligament injection" AND "Postendodontic pain" OR "Postobturation pain"
Web of Science	TS="Root canal treatment" OR "Endodontic treatment" AND TS="Preemptive corticosteroids" OR "Parenteral administration" AND TS="Postendodontic pain" OR "Postobturation pain"

1

**Table 2:** General information of included article.

Author and year	Sample size	Age	Pre operative status	Study groups	Volume of the corticosteroid	Mode of corticosteroid administration	Type of syringe/needle	Initial administration of Local anesthesia
Kaufman et al. 199413	45	19-71 years	Irreversible pulpitis, pulpal necrosis with apical periodontitis.	Group 1: slow-release methylprednisolone intraligamentary Group 2: active placebo drug, mepivacaine 3% Group 3: local anesthesia, passive placebo.	4 single rooted to 8 mg multi rooted	Group 1 and 2 intraligamentary administration	stainless steel carpule to fit the intraligamentary syringe Ligmaject IMAAssociates, Cottrele and Co., London, Wise.	Initial administration not reported
Mehrvarzfar et al. 200814	100	21-58 years	Irreversible pulpitis	Group 1: 4 mg of the active drug dexamethasone infiltrationGroup 2 received lidocaine 2% as a placebo infiltration	1ml	Infiltrative	Not reported	Initial administration not reported
Sharma et al. 201515	100	18-50	Irreversible pulpitis	Group 1: PlaceboGroup 2: Dexamethasone Subgroup 1: oralSubgroup 2: intramuscularSubgroup 3: infiltrationSubgroup 4:intraligamentary	Not mentioned	Intramuscular, infiltration and intraligamentary	Not mentioned	2% Lidocaine in 1:100,000 epinephrine
Mehrvarzfar et al. 201616	60	18-65	Symptomatic irreversible pulpitis	Group 1 – Intraligamnetary injection with an empty cartridgeGroup 2: 2% lidocaine intraligamnetary InjectionGroup 3:Dexamethasone intraligamnetary injection	Group 2 and 3- 0.2ml	Intraligamnetary	30-gauge needle secured to an intra-ligamental syringe.	2% lidocainebuccal inflitration for maxillary molars and IANB for mandibular molars
Aksoy et al. 202017	90	18-65	Symptomatic irreversible pulpitis	Group 1: saline infiltration control groupGroup 2: tramadol infiltration Abdi Ä°brahim Pharmaceutical Co., Istanbul,Turkey andGroup 3: Submucosal dexamethasone infiltration Deva Co., Istanbul, Turkey	Group 1: 2ml Group 2: 100 mg/2 mLGroup 3: 8 mg/2 mL	Infiltrative	27-gauge needles.	4% articaine with 1:200,000 epinephrine IANB block
Yavari HR et al. 201918	242	20-50	Irreversible pulpitis	Group 1: dexamethsone infiltrationGroup 2: betamethasone infiltrationGroup 3: sterile saline infiltration	Group 1, 2, 3: 0.7ml	Infiltration	Infiltration of 0.7ml of test agent	1.5ml of lidocaine 1:100000 epinephrine
Shekar RM et al. 202419	90	20-50	Symptomatic irreversible pulpitis	Group 1: Intramuscular saline injection PlaceboGroup 2: 4mg dexamethasone intramuscular injectionGroup 3: 30mg ketorolac intramuscular injection	Group 1: 2mlGroup 2: 4mg/2mlGroup 3: 30mg/2ml	Intramuscular injection	Intramuscular injection of 2ml solution at the deltoid muscle site using a 25-gauge, 16 mm needle.	2% lidocaine
Shantiaee Y et al. 201220	90	18-42	Irreversible pulpitis or apical periodontitis	Group 1: Normal saline infiltrationGroup 2: dexamethasone infiltrationGroup 3: Morphine infiltration	Group 1: 1mlGroup 2: 4mg/mlGroup 3: 10mg/ml	Infiltration	Not mentioned	Unclear
Fatima S et al. 202521	100	18-65	Symptomatic apical periodontitis	Group 1: Control group PlaceboGroup 2: Intraligamnetary dexamethasone group	Group 2: 0.2ml of dexamethasone	Intraligamnetary	Not mentioned	1.8ml of 2% lidocaine containing 1:80,000 epinephrine

2

**Table 3:** Evaluation criteria.

Author and year	Pre operative and postoperative pain evaluation	Time interval	Analgesic intake	Results																		Outcome
Kaufman et al. 199413	1-10 visual analogue scale	24 hours	Analgesics consumption noted 3 patients in the group 1 reported postoperative pain had taken self-prescribed analgesic	Group					Post operative pain Mean							Standard error						Significant decrease in postoperative pain in methylprednisolone group than placebo groups. 22% in the methylprednisolone group reported with pain than 76% and 50% in active and passive placebo groups.
				Methylprednisolone					0.385							0.213						
				Mepivacaine					2.471							0.570						
				No drug					2.700							1.023						
Mehrvarzfar et al. 200814	0-9 visual analogue scalemodified by DrTorabinejad	6, 12, 24 and 48 hours	Analgesic taken patients were excluded	Time period					Placebo % pain							Dexamethasone % pain						Dexamethasone associated with less frequent pain at 6, 12 and 24 hours. No difference between two groups after 48 hours.
				Postoperative pain at 6 h					40							8						
				Postoperative pain at 12 h					34							2						
				Postoperative pain at 24 h					24							6						
				Postoperative pain at 48 h					8							8						
Sharma et al. 2015 15	10 point visual analogue scale	6, 12, and 24 hours	Acetaminophen	Time period		Placebo% pain			Oral dexamethasone% pain		Intramuscular dexamethasone% pain					Intraligamentary dexamethasone % pain					Infiltrative dexamethasone % pain	Oral route reported less pain than intraligamentary at 6, 12 and 24 hours. Significant difference found between intramuscular and supraperiosteal at 6 hour.Supraperiosteal reported higher pain reduction than intramuscular followed by intraligamentary but difference between supraperiosteal and intraligamentary was significant only at 12 and 24 hour interval
				6 h		65.1			83.6		68.7					56.2					61.8	
				12 h		59			89		85.3					74.1					61.3	
				24 h		61.7			89.5		84.6					78.7					63.4	
Mehrvarzfar et al. 2016 16	170 mm visual analog scale	6, 12, 24 and 48 hours	6 hours- placebo 70%, lidocaine 50%, dexamethasone 40%. 12, 24 hours- Placebo 60%, lidocaine 25% dexamethasone 25%. 48 hours- no analgesic consumption	Time period				Placebo						Lidocaine					Dexamethasone			Pain reduction was better with dexamethasone group.12 hour pain intensity was least with dexamethasone. 24 and 48hour, placebo and dexamethasone group reported the more pain respectively& difference was not significant with others.
				Before treatment				106.4 35.4						97.8 036.07					100.6 29.61			
				Postoperative pain at 6 h				80 44.6						50.45 26.9					35.25 17.47			
				Postoperative pain at 12 h				45 30.3						30.05 17.06					12.3 35.4			
				Postoperative pain at 24 h				14 12.8						11.85 10.76					7.7 9.91			
Aksoy et al. 2020 17	170mm Heft Parker VisualAnalogue Scale	6, 12, 24, 48 and 72 hours	400 mg ibuprofen 6hour- 7 patients in control group, 4 in group 2 and 3 in the group 3 12 h, 10 patients in control group, 3 in the group 2 and 1 in group 3	Time period				Control						Tramadol			Dexamethasone					6, 12, 24 and 48 hour pain intensity were less with dexamethasone and tramadol, with no elicited difference at 72 hour interval.
				Preoperative pain				109.33±9.35						106.10±13.80			108.87±13.81					
				Postoperative pain at 6 h				9030-125						45 20-95			32 0-85					
				Postoperative pain at 12 h				56 10-80						38 10-60			20 0-75					
				Postoperative pain at 24 h				35 10-65						22.5 0-50			18 0-50					
				Postoperative pain at 48 h				21 0-50						10 0-30			10 0-40					
				Postoperative pain at 72 h				5 0-35						2,5 0-20			1 0-20					
Yavari HR et al., 2019 18	visual analogue scale	6, 12, 24, 48, 72 hour and 7-day intervals	Not mentioned	Time period			Placebo					Dexamethasone							Betamethasone			Pain intensity significantly decreased in all groups post-treatment, but the Dexamethasone group consistently showed the lowest pain scores at all time intervals, with a marked reduction from 5.21 before treatment to 0.64 at 7 days P < .001. The Long-acting Betamethasone group demonstrated better pain control than the Placebo group, especially at 48 hours 1.92 ± 1.85 and 72 hours 0.25 ± 0.66, indicating its prolonged analgesic effect. The Placebo group showed significantly higher pain scores than both steroid groups at all post-treatment time points P < .001, confirming the superior efficacy of corticosteroids in pain management.
				Before treatment			5.23					5.21							5.29			
				Postoperative pain at 4 h			2.13					0.75							1.68			
				Postoperative pain at 12 h			4.16					1.57							3.42			
				Postoperative pain at 24 h			4.00					1.79							3.08			
				Postoperative pain at 48 h			3.80					2.06							1.92			
				Postoperative pain at 72 hours			1.59					0.82							0.25			
				Postoperative pain at 7 days			0.93					0.64								0.187		
Shekar RM et al. 2024 19	Verbal rating scale	24 and 48 hours	400 mg ibuprofen	Group	Pre-treatment Mean ± SD					4th hr Mean ± SD					24th hr Mean ± SD					48th hr Mean ± SD		Pain scores sigificantly decreased in all groups post-treatment, with the Dexamethasone and Ketorolac Tromethamine groups showing lower pain levels compared to the Placebo group at all time intervals. The Dexamethasone group exhibited the most consistent reduction in pain from the 4th hour to the 48th hour, with mean scores dropping from 1.2 ± 0.3 to 1.0 ± 0.2. The Placebo group demonstrated higher pain scores at the 4th hour 2.0 ± 0.4 and 24th hour 1.5 ± 0.3 compared to both active treatment groups, indicating the limited effect of placebo intervention.
				Dexamethasone Group	3.5 ± 0.5					1.2 ± 0.3					1.1 ± 0.2					1.0 ± 0.2		
				Ketorolac Tromethamine Group	3.5 ± 0.6					1.1 ± 0.4					1.3 ± 0.4					1.0 ± 0.3		
				Placebo Group	3.5 ± 0.6					2.0 ± 0.4					1.5 ± 0.3					1.2 ± 0.3		
Shantiaee Y et al. 2012 20	visual analogue scale	4, 8, 24, 48 hours	500 mg acetaminophen	Time period				Placebo					Dexamethasone					Morphine				Pain intensity was consistently higher in the Placebo group at all time intervals compared to both Dexamethasone and Morphine groups, indicating the effectiveness of active treatments in pain reduction. The Dexamethasone group showed the lowest pain scores across all time points, with a gradual decline from 2 at 4 hours to 1.7 at 48 hours, suggesting its sustained analgesic effect. Although Morphine provided better pain relief than placebo, its pain scores were higher than Dexamethasone at all time intervals except 48 hours, highlighting Dexamethasone as a more potent drug.
				Postoperative pain at 4 h				5.9					2					2.3				
				Postoperative pain at 8 h				5.85					1.96					2.2				
				Postoperative pain at 24 h				5.2					1.8					2				
				Postoperative pain at 48 h				3.2					1.7					2.65				

3

## Data Availability

The datasets used and/or analyzed during the current study are available from the corresponding author.
